# Intelligent Monitoring of Care Status for COPD Patients Based on Deep Learning

**DOI:** 10.1155/2021/5690442

**Published:** 2021-11-22

**Authors:** Xiaoqun Chen, Yufen Yao

**Affiliations:** Respiratory Medicine, Zhejiang Hospital, Hangzhou, Zhejiang 310013, China

## Abstract

To discuss the application method and effect of COPD patients in deep learning in intelligent monitoring, two groups were used under a reasonable selection of antibiotics specifically including reasonable and effective oxygen administration, atomization, sputum discharge treatment, psychotherapy, and rehabilitation training and treatment. Results were indicated, and there were significant differences between the lung function evaluation index and the two groups. Its intelligent monitoring mode was 97.5% and 80.0%, while the red blood cell ratio, arterial oxygen partial pressure (PaO_2_), pulse blood oxygen saturation (SpO_2_), arterial carbon dioxide partial pressure (PaCO_2_), and symptom improvement were better than artificial and were statistically significant (*P* < 0.05). Therefore, the training of the anti-inspiratory muscle can effectively improve the lung function and dyspnea symptoms of COPD patients at the stable stage, thus greatly improving their respiratory function and ensuring the quality of life of patients, which is worthy of clinical application.

## 1. Introduction

Chronic obstructive pulmonary disease (COPD) is a relatively common chronic disease in respiratory and critical care medicine. And sleep apnea-hypopnea syndrome (SAHS) is also known as overlap syndrome. When both exist together, they are one of the reasons for recurrent severe hypoxemia at night in COPD patients [1]. According to the condition of hypoxic COPD patients at night, we take corresponding nursing measures for COPD patients with great physical and mental harm. It belongs to airway obstructive disease with many pathological factors such as environmental and genetic; clinical manifestations are diverse, causing obstacles to normal breathing, seriously affecting the quality of life of patients, with a high mortality rate. Choosing an effective treatment is the key to improving prognosis, and the most common symptoms and features of the disease are persistent respiratory symptoms and restricted airflow.

Chronic obstructive pulmonary disease is defined in the GOLD guidelines as usually caused by abnormal airway and/or alveoli resulting from significant long-term exposure to toxic gases or particles. Since airway pathological changes in COPD patients are accumulated over a long period of time, they are more frequent in middle-aged and elderly people. In the later stage, with the progression of the disease and the onset of acute exacerbation of the disease, the chronic course of the disease has a serious impact on the quality of life of the patients [2]. Moreover, the morbidity and mortality of the disease remain high, and the economic and social burden of the disease is heavy, as confirmed by China's National Statistical Yearbook. COPD is deformed by respiratory obstruction. In the calm state, the body's oxygen consumption level is low, and there may be no obvious symptoms. When the body's oxygen consumption increases, the patient may have difficulty breathing and other symptoms. At present, lung function examination is an internationally recognized important method to diagnose and evaluate the severity of COPD, and the evaluation indexes for the severity of pulmonary function of COPD clearly include forced expiratory volume (FEV1) and vital capacity (FVC) [3]. Lung rehabilitation training has been proved to be an effective treatment for COPD patients both in the COPD guidelines and in scientific studies at home and abroad, and it is a more favorable method for the recovery of lung function and disease stability.

Based on this, it adopts the method of intelligent antiobstructive inspiratory muscle, which can help to reduce the degree of dyspnea of patients, promote recovery as soon as possible, and effectively increase the lung capacity through training, which is convenient for the same method of training to be widely carried out in clinical treatment. The results of this study show that the intervention effect of intelligent antiobstructive inspiratory muscle training is significantly better than ordinary pulmonary rehabilitation breathing training for COPD patients in the stable stage, indicating that intelligent antiobstructive inspiratory muscle training does have a significant effect on the intervention of COPD patients in the stable stage. It is worthy of future clinical application [4].

## 2. Methods of Study

### 2.1. Clinical Data

A total of 122 COPD stable patients in a hospital were selected. The inclusion criteria were as follows: ① meet the diagnostic criteria of COPD; ② in the stable stage of disease and standardized drug treatment; ③ clear, no communication barriers; ④ there is no possibility of pneumothorax and spontaneous rib fracture. The exclusion criteria were as follows: ① COPD acute exacerbation, the condition is not stable; (2) cognitive impairment cannot cooperate with treatment; ③ complicated with serious heart, brain, and lung diseases; ④ serious organ injury; ⑤ other patients with respiratory airflow restriction diseases. Patients were randomly divided into the intelligent group and artificial group, 61 cases in each group. In the intelligent group, 31 males and 30 females were aged from 42 to 74 years. The education level was as follows: 31 cases had no education experience, 20 cases were middle school and high school, and 10 cases were high school or above. In the artificial group, there were 30 males and 31 females, aged from 40 to 73 years; the education level was as follows: 30 cases had no education experience, 21 cases were junior high school and senior high school, and 10 cases were senior high school or above. There was no significant difference in general data between the two groups (*P* > 0.05). There was no significant difference between the intelligent group and artificial group in gender, age, disease, smoking history, educational background, and social experience, and *P* > 0.05, indicating that the general data of patients in the two groups were clinically comparable [5].

### 2.2. Artificial Law

Traditional pulmonary rehabilitation breathing training includes abdominal breathing, controlled deep breathing, and lip contraction breathing. ① Abdominal breathing, also known as diaphragmatic breathing, refers to any method based on diaphragmatic breathing in clinic or life. Abdominal breathing training can effectively help increase the activity of the diaphragmatic muscles and minimize the activity of the auxiliary breathing muscles, thus reducing the respiratory rate and increasing the minute ventilation [6]. Abdominal breathing training methods are as follows: researchers to assist the patient to sitting and lying positions or semifowler, relax the whole body, the left hand on the chest, right hand on your abdomen navel position, keeping the chest, inhale, feel belly up, exhale, and feel abdomen decline; in this cycle training, keep the agreement of the rhythm of every breath [7]. Abdominal breathing training is conducted twice at 9 : 30 and 15 : 30 every day for about 10 minutes. The key to abdominal breathing is exhale and inhale as much as possible to carry out abdominal movement in order to maximize the diaphragm movement and enhance the effect of exercise. ② Push lip breathing: this method prevents small airways from collapsing prematurely, helping to drain the remaining air from the lungs to the maximum. Specific methods: when the patient carries out abdominal breathing, the lips should breathe in the shape of whistle, and the size of lip gap should be suitable for the patient to feel comfortable. During breathing, the candle flame in front of the lips should be inclined by 15 degrees, and the ratio of inspiratory and expiratory time is 1 : 2 or 1 : 3 ③ Controlled deep breathing: it refers to that patients consciously take controlled deep and slow breathing, pause at the end of inspiratory for 1–3 s, and then breathe again. Patients deliberately control the length of inspiratory and exhalation time with autonomous consciousness and deliberately control the frequency of trained breathing, the depth, and the position of breathing movement [8–10].

#### 2.2.1. Intelligent Inspiratory Muscle Training Method

**Figure 1:**
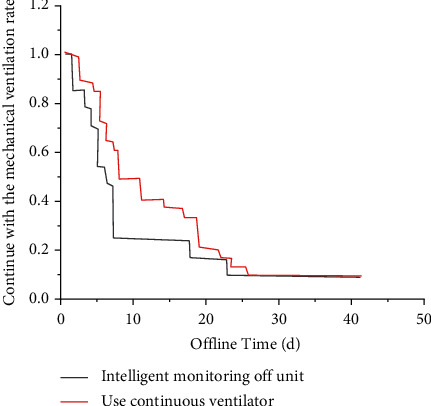
Kaplan–Meier curves of shedding probability of two offline methods.

Based on traditional pulmonary rehabilitation breathing training, progressive intelligent resistance inspiratory muscle training was carried out. Studies have proved that the inspiratory muscle can improve its strength and endurance through muscle strength training. The best effect can be achieved by increasing appropriate resistance load in inspiratory resistance training. Antibiotics were reasonably selected in both groups, and sensitive antibiotics were selected according to the drug sensitivity test results of COPD patients combined with specific conditions, and sufficient course of treatment and sufficient amount of treatment were performed. When patients' condition was mild or moderate, the first or second generation of cephalosporins, such as cefuroxime, was given. Patients with severe conditions can be given third-generation cephalosporins, such as ceftriaxone. In the course of treatment, cell load is reduced to the lowest level as far as possible to effectively prevent fungal infection. For example, on the basis of the above, the intelligent group of salbutamol was treated with comprehensive means, including reasonable and effective oxygenation, atomization and expectoration therapy, psychological therapy, and rehabilitation training therapy [11]. Then, the specific volume of red blood cells, arterial partial pressure of oxygen (PaO_2_), pulse oxygen saturation (SpO_2_), arterial partial pressure of carbon dioxide (PaCO_2_), and improvement of symptoms of the two patients were observed and recorded for statistical analysis, and the conclusions were drawn, as shown in Figure 1.

### 2.3. COPD Is due to Its High Incidence, Heavy Burden of the Disease, and High Fatality Rate

In the clinical treatment of COPD, drug therapy alone can not effectively prevent the process of disease. According to the characteristics of the patient's condition, comprehensive treatment can significantly alleviate the clinical symptoms, so as to improve the patient's alveolar ventilation function. The condition can be stabilized, and the progress of the disease to delay the effect greatly improves the quality of life of patients. COPD patients are mostly elderly people, whose stress ability is weakened, their immune function is decreased, their sensitivity to antibiotics is reduced, their physique is poor, and they usually suffer from basic diseases such as coronary heart disease and diabetes; according to the results of drug sensitivity, the selection of antibiotics is the key to make the disease get good treatment. In this study, in addition to drug therapy, the intelligent group also included the application of oxidation, atomization inhalation, rehabilitation training, psychological therapy, and other comprehensive interventions, with satisfactory clinical effects.

#### 2.3.1. Oxygen Therapy

Oxygen supply is effective and reasonable, using more than 15 h/d intermittent oxygen inhalation, keep oxygen flow in 2-3 L/min, maintain oxygen concentration in 25%–29% which is appropriate, and strengthen the control of the oxygen humidity value in the process of oxygen inhalation. At the same time, the patient's vital signs and disease changes were closely observed during oxygen therapy.

#### 2.3.2. Fog Inhalation

Keep the respiratory tract unobstructed, teach patients effective expectoration methods, and encourage active expectoration. Patients with dry sputum, sticky sputum, and difficult cough can be treated with aerosol inhalation, such as ambroxol 30 mg and terbutaline sulfate 0.25 mg, 2 times/d, to dilute sputum, and create conditions for expectoration. For patients who are unable to expectorate, effective measures can be taken for sputum aspiration, tracheotomy, or endotracheal intubation and assisted mechanical respiratory therapy if necessary [12–14].

#### 2.3.3. Rehabilitation Training

Abdominal breathing muscle exercise is used to guide patients, and lip breathing is carried out at the same time, so as to enhance the endurance and muscle strength of chest respiratory muscle and diaphragm respiratory muscle, and gradually carry out rehabilitation training to promote the rehabilitation of patients. At the same time, guide the diet, mainly light diet.

#### 2.3.4. Mind Cure

COPD patients have a long course of disease, serious condition, and are easy to relapse. COPD will have a serious impact on the quality of life of patients. Patients are easy to have negative emotions such as anxiety, depression, and pessimism. Nurses need to actively communicate with patients and instill relevant knowledge and methods of the necessity, advantages, precautions, and long-term efficacy of disease treatment. Successful cases of the same type of treatment are introduced to make patients have confidence in fighting against the disease, eliminate ideological concerns, and cooperate with treatment actively [15].

#### 2.3.5. Effect Assessment

According to the standards of relevant departments, the clinical effect was evaluated, and the clinical control was as follows: lung function was improved at grade 2 or above, and cough, asthma, sputum, and pulmonary sound were restored to the preacute exacerbation level; effective: lung function improved at least grade 1, and cough, asthma, sputum, and lung sound improved, but did not return to the level before acute exacerbation; ineffective: exacerbation of the disease and no improvement in pulmonary sound, cough, asthma, and sputum [16, 17]. At the same time, the changes of arterial blood gas analysis and erythrocyte volume after treatment were recorded in the two groups, and the improvement time of clinical symptoms was compared.

#### 2.3.6. Statistical Methods

Statistical software SPSS 13.0 was used, measurement data were expressed as mean ± standard deviation ($\bar{x}\pm s$), and *t*-test was used. The percentage of counting data was expressed by *X*^2^ test. *P* < 0.05 was considered statistically significant.

### 2.4. COPD Is the Result of Multiple Molecular Genetic Factors and Pathological and Clinical Factors

Most COPD patients are accustomed to rely on drug therapy but ignore the importance of pulmonary rehabilitation in the course of the COPD disease. Many scholars at home and abroad believe that pulmonary rehabilitation therapy is one of the most important methods in clinical stable COPD patients besides drug therapy. COPD testing reports suggest that almost all COPD patients can benefit from regular pulmonary rehabilitation training during the stable phase of the disease. It has been reported that pulmonary rehabilitation treatment in stable COPD patients can largely improve the lung function of patients, relieve the symptoms of dyspnea, and then improve the prognosis of the disease. The intervention effect of intelligent antiobstructive inspiratory muscle training for COPD patients in the stable stage is significantly better than that of ordinary pulmonary rehabilitation breathing training, indicating that intelligent antiobstructive inspiratory muscle training does have a significant effect on COPD patients in the stable stage.

## 3. Results' Analysis

It can be seen from Tables 1 and 2 that there are statistically significant differences in scores. The total effective rate of the intelligent monitoring mode was 97.5%, and the total effective rate of the artificial monitoring mode was 80.0%. The changes of RBC, arterial partial pressure of oxygen (PaO_2_), pulse oxygen saturation (SpO_2_), arterial partial pressure of carbon dioxide (PaCO_2_), and symptom improvement after treatment in the intelligent monitoring mode were better than those in the artificial monitoring mode. The difference was statistically significant (*P* < 0.05).

## 4. Discussion

### 4.1. The Main Manifestations of COPD Are Emphysema and/or Chronic Bronchitis

Dyspnea, shortness of breath, expectoration, cough, and other symptoms are more frequent and common in the clinic of the disease. The main clinical feature of the disease is airflow obstruction, which induces airway obstruction in patients, and the course of the disease will gradually deepen. Some patients may be accompanied by airway hyperresponsiveness. The disease is usually characterized by airflow obstruction, reversibility, and airway hyperresponsiveness. As a disease with obvious pathological changes in broncholung tissues and severe decline in lung function, this disease often causes great interference to the quality of life of patients, and drug therapy alone is often difficult to achieve ideal efficacy. Therefore, we implemented this treatment for observation, as shown in Tables 3 and 4.

According to the data in Tables 3 and 4, after the effective treatment, the total effective rate was as high as 93.33%, and the blood gas analysis results of the patients were also improved (*P* < 0.05), indicating that the treatment was effective.

We analyze the reasons as follows: (1) psychological disorder counseling: because of the characteristics of chronic obstructive pulmonary disease (long course of the disease, serious condition, and easy to repeat) and the population of the disease is the elderly, such patients are prone to adverse psychological problems such as depression, pessimism and disappointment, anxiety, fear, and nervousness. According to the situation, we have adopted various ways and forms to strengthen the publicity of chronic obstructive pulmonary disease. And we strengthened the communication with patients, as well as the explanation of disease knowledge and successful cases (attitude should be paid attention to this kind, especially for patients with physical defects and personal privacy protective language), and helped the elderly patients through mental difficulty. ② To keep the respiratory tract unobstructed: first of all, patients are encouraged to cough actively. Secondly, 0.25 mg terbutaline sulfate and 1mg budesonide were used for atomization inhalation twice a day to treat symptoms such as viscous sputum and not easy to cough up. The patients with weak expectoration should be treated with sputum aspiration and intubation when necessary. ③ Actively improve the respiratory function of patients under the condition of unimpeded respiratory tract, such as intermittent oxygen (10–12 h/d), oxygen flow at 2-3 l/min, and concentration maintained at 25%–29%; at the same time, patients were instructed to perform lip contraction and exhalation, abdominal breathing muscle training, etc., so as to improve the strength and endurance of respiratory muscles and improve respiratory function. (4) Drug treatment: for example, antibiotics are selected and used in combination. Therefore, we often select highly sensitive antibiotics according to the patient's condition and drug sensitivity results and ensure sufficient quantity and course of treatment. For example, the first- and second-generation cephalosporins can be selected for mild or moderate patients, while the third-generation cephalosporins are selected for severe patients. At the same time, pay attention to reduce the incidence of fungal infection during treatment; in addition, rational use of bronchodilators such as salbutamol can effectively inhibit airway submucosal inflammation, relax the bronchial smooth muscle, eliminate luminal secretions, and inhibit airway edema caused by endogenous media. In conclusion, strengthening the prevention and treatment of COPD has inestimable clinical value in improving patients' cardiopulmonary function, effectively reducing the recurrence rate, and improving patients' quality of life [18, 19].

## 5. Conclusion

In conclusion, COPD patients can be treated with antibiotics combined with reasonable and effective oxygen inhalation, atomization and sputum treatment, psychotherapy, rehabilitation training, and other comprehensive methods. It can significantly improve the clinical treatment effect of patients, erythrocyte-specific volume, arterial partial pressure of oxygen (PaO_2_), pulse oxygen saturation (SpO_2_), symptom improvement, and other changes after treatment so that the respiratory function has been greatly improved to ensure the quality of life of patients. Its intelligent antiobstructive inspiratory muscle training method also has a significant effect on the intervention of COPD patients at the stable stage. The intelligent antiobstructive inspiratory muscle method is adopted, which can help to reduce the degree of dyspnea of patients and promote recovery as soon as possible. Through training, lung capacity was increased effectively, which was convenient for the same training to be widely carried out in clinical treatment. The results of this study show that the intervention effect of intelligent antiobstructive inspiratory muscle training is significantly better than that of ordinary pulmonary rehabilitation breathing training for COPD patients in the stable stage, indicating that intelligent antiobstructive inspiratory muscle training does have a significant effect on the intervention of COPD patients in the stable stage. It is worthy of future clinical application.

## Figures and Tables

**Table 1:** Proportion of the clinical therapeutic effect between the two groups of patients with chronic obstructive pulmonary disease (%).

Group	Example number	Clinical control	Effective	Invalid	Efficiency (%)
Intelligent group	40	32 (80.0)	7 (17.5)	1 (2.5)	97.5
Artificial group	40	23 (57.5)	9 (22.5)	8 (20.0)	80.0

Compared with the manual, *P* < 0.05.

**Table 2:** Comparison of indicators between the two groups of patients with COPD after treatment ($\bar{x}\pm s$).

Group	Example number	SpO_2_ (%)	PaO_2_ (mmHg)	PaCO_2_ (mmHg)	Erythrocrit	Symptoms' improvement (d)
Intelligent group	40	93.0 ± 2.5	82 ± 4	42.0 ± 2.5	0.47 ± 0.07	4.3 ± 3.2
Artificial group	40	88.0 ± 4.1	75 ± 3.2	46.0 ± 3.3	0.62 ± 0.07	8.6 ± 3.2

Compared with the manual period, *P* < 0.05.

**Table 3:** Clinical therapeutic effect of chronic obstructive pulmonary disease.

Clinical effects	Clinical control	Excellence	Effective	Invalid	Total effective
Example number (*n*)	9	14	5	2	28
Scale (%)	30.00	46.67	16.67	6.67	93.33

**Table 4:** Blood gas analysis results of chronic obstructive pulmonary disease patients.

Time	Example number	Partial pressure of carbon dioxide	Oxygen compression
Pretherapy	30	52.00 ± 5.00	85.60 ± 2.60
Posttreatment	30	34.80 ± 2.20	92.00 ± 2.20

## Data Availability

The data used to support the findings of this study are available from the corresponding author upon request.
